# Standardized phylogenetic and molecular evolutionary analysis applied to species across the microbial tree of life

**DOI:** 10.1038/s41598-020-58356-1

**Published:** 2020-02-03

**Authors:** Migun Shakya, Sanaa A. Ahmed, Karen W. Davenport, Mark C. Flynn, Chien-Chi Lo, Patrick S. G. Chain

**Affiliations:** Bioscience Division, Los Alamos National Laboratory, MS-M888, Los Alamos, NM 87545 USA

**Keywords:** Data processing, Genome informatics, Phylogeny, Software

## Abstract

There is growing interest in reconstructing phylogenies from the copious amounts of genome sequencing projects that target related viral, bacterial or eukaryotic organisms. To facilitate the construction of standardized and robust phylogenies for disparate types of projects, we have developed a complete bioinformatic workflow, with a web-based component to perform phylogenetic and molecular evolutionary (PhaME) analysis from sequencing reads, draft assemblies or completed genomes of closely related organisms. Furthermore, the ability to incorporate raw data, including some metagenomic samples containing a target organism (e.g. from clinical samples with suspected infectious agents), shows promise for the rapid phylogenetic characterization of organisms within complex samples without the need for prior assembly.

## Introduction

The reconstruction of organismal evolutionary history using phylogenetics is a fundamental method applied to many areas of biology. Single nucleotide polymorphisms (SNPs), one of the dominant forms of evolutionary change, have become an indispensable tool for phylogenetic analyses^1–4^. Phylogenies in the pre-genomic era relied on SNPs and conserved sites within a single locus, and was later extended to multiple loci, such as in multiple locus sequence typing (MLST). Although still valuable, these methods only consider evolutionary signals originating within a small fraction of the genome, are unable to capture the complete variation within species, and generally provide a weak phylogenetic signal, particularly within a species, and do not always reflect the true evolutionary history of species^5^. While phylogenetic analyses that use many conserved genes (orthologs) are a great improvement, these methods require annotated coding regions, whose predictions are not always accurate or available^6^. Furthermore, they are impacted by horizontal gene transfer (HGT)^7^, recombination^8^, rate heterogeneity^9^, and incomplete lineage sorting.

Genome-wide SNPs are one of the best measures of phylogenetic diversity as they can discriminate among closely related organisms and help resolve both short and long branches in a tree^10,11^. Since selectively neutral SNPs accumulate at a uniform rate, they can be used to measure divergence between species as well as strains^12,13^. Furthermore, due to the large number of SNPs found along the length of entire genomes, the use of whole-genome SNPs minimizes the impact of random sequencing and assembly errors that can impact individual loci, as well as biases due to individual genes under strong selective pressure. Some inherent biases remain with whole genome SNP approaches that are similar to loci-based phylogenies such as HGT, recombination, and rate heterogeneity. Although genome-wide sequencing now allows examination of the full complement of genomic variation, the number of completed and finished genomes are increasingly falling behind the generation of new draft genomes, due to the lack of computational or other resources. For example, of 94,126 total genomes in the NCBI RefSeq genome database, only 13.25% are complete (December 5, 2018 from ftp://ftp.ncbi.nlm.nih.gov/genomes/refseq/assembly_summary_refseq.txt) and a large fraction of available sequencing data still remains unassembled as evident by the much larger number (e.g. 360,929 for bacteria only) of whole genome projects in the sequence read archive (SRA) database (June 21, 2018 from https://www.ncbi.nlm.nih.gov/sra/). Several methods for whole-genome SNP discovery or phylogenetics have been previously described: SNPsFinder^14^, PhyloSNP^2^, kSNP^15^, WG-FAST^16^, NASP^17^, CFSAN^18^, CSI phylogeny^19^, REALPHY^20^, SNVPhyl^21^, SPANDx^22^, Snippy^23^, Lyve-set^24^, and Parsnp^25^. Some of these (e.g. SNPsFinder and PhyloSNP) are no longer under active development. Although the others are able to analyze raw reads to identify a core genome (the conserved portion among all genomes) and the SNPs within it, several of them cannot process assembled contigs or multiple complete genomes (e.g., CFSAN, SPANDx, Lyve-set), or will perform only a portion of the required functions to obtain a tree (e.g., Snippy), or identify SNPs from metagenomes (e.g. WG-FAST), and only few (CSI Phylogeny, REALPHY, SNVPhyl) can be accessed with a graphical user interface, limiting the user base to well-trained bioinformatics scientists. Moreover, almost all these tools have been restricted in their testing to bacterial organisms, and have only been used with genomes from within a single species. None have shown broad utility incorporating multiple species (i.e. genus-level phylogeny) or genera within a single tree, nor have any been tested on microbial eukaryotic genomes. In addition, most of these tools require users to select a reference genome, which can have dramatic impact on the alignments and resulting SNP calls^26^, and are unable to distinguish or map SNPs to their functional annotation, and hence cannot perform molecular evolution analysis.

**Figure 1:**
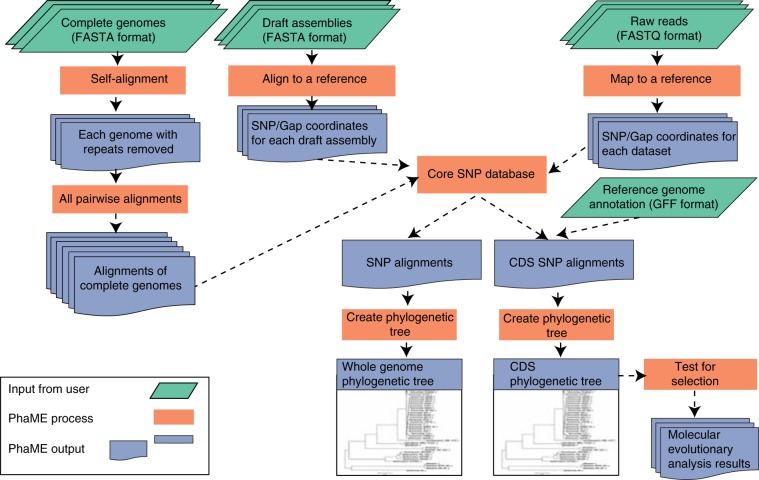
PhaME analysis workflow. The PhaME analysis workflow first identifies SNPs at orthologous positions in complete genomes, assembled contigs, and read datasets. First, nucmer is used to identify and mask repeats, and to perform pairwise alignments among all complete genomes. A reference genome is selected based on user criteria (See Methods). Contigs are then compared with the reference genome using nucmer, and reads are then mapped to the reference using Bowtie 2 or BWA. The SNP and gap coordinates are used to generate whole-genome core alignment. If an annotation file is provided, a separate alignment consisting of conserved positions only found in the CDS regions are also reported. RAxML, FastTree or IQ-TREE phylogenies are constructed using these alignments. If specified, PAML or HyPhy packages are used to test for selective pressure on genes with SNPs.

Here, we present an open source workflow using a collection of existing bioinformatic tools for Phylogenetic and Molecular Evolutionary (PhaME) analysis that incorporates these additional features to allow more flexibility when studying the evolutionary relationships between closely related genomes (genera, species, and strains). PhaME is a whole-genome SNP-based phylogeny tool that identifies the core genome from input datasets (finished genomes, draft assembly contigs, and/or raw FASTQ reads), extracts core SNPs, parses them to coding or non-coding regions and as synonymous or non-synonymous SNPs, reconstructs a phylogeny, and performs molecular evolutionary analysis to identify genes under selection (Fig. 1). With any of the inputs there must be sufficient data covering a target genome of interest for acceptable SNP calling. PhaME thus accepts FASTA or FASTQ inputs corresponding either to genome sequencing data from isolates, or metagenomic data where the target organism has sufficient reads to allow SNP calling along much of the length of the genome. PhaME can be run either via the command line, or accessed through an accompanying webserver that can be installed locally. Here, we demonstrate PhaME’s ability to construct robust genus and species phylogenies using examples that span the tree of life, with up to thousands of genomes as input in the form of raw sequencing reads, draft assembled contigs, fully completed genomes, and even unassembled metagenomic reads.

## Results and Discussions

### Implementation of PhaME with examples from across the tree of life

To demonstrate different capabilities of PhaME and to validate the underlying algorithms, we tested PhaME on available bacterial genomes of *Escherichia* (together with related genera *Shigella*, and *Salmonella*) and *Burkholderia* (together with recently reclassified genera of *Caballeronia* and *Paraburkholderia*, and *Ralstonia* as an outgroup), as well as on eukaryotic genomes from *Saccharomyces*, and on viral genomes of *Zaire ebolavirus*. We further examined the robustness of how PhaME handles raw reads, by comparing the placement of these datasets with the genome assemblies that resulted from these data, and have also investigated how well PhaME performs when including metagenomic samples in the form of raw reads (Table 1).

**Table 1:** Summary statistics of PhaME Analyses.

	#of genomes (complete/assemblies/reads)	Average genome size*	Core genome Size**	% core	Core SNPs***	% Core SNPs	CDS SNPs^¶^	% CDS SNPs
Escherichia *and* Shigella	35/0/0	5,078,265	2,159,296	42.5	266,969	12.4	248,243	93.0
Escherichia (Shigella*) and* Salmonella	630/46/0	4,949,086	134,062	2.7	40,675	30.3	39,201	96.4
Burkholderia, Paraburkholderia*, and* Cabelleronia	70/88/55	7,303,088	43,124	0.6	15,180	35.2	15,152	99.8
Burkholderia pseudomallei/mallei	26/36/32	6,964,607	2,802,743	40.2	756,597	27.0	686,400	90.7
Bcc	16/18/10	7,525,439	699,313	9.3	97,524	14.0	94,076	96.5
Saccharomyces	2/185/7	11,413,241	96,665	0.85	24,244	25.1	23,330	96.2
S. cerevisiae	2/164/6	12,088,140	2,224,283	18.4	543,865	24.5	456,488	83.9
Zaire ebolavirus^#^	1359/0/0	18,812	17,639	93.8	1,787	10.1	NA	NA
Zaire ebolavirus *(Sierra Leone)*	938/0/93	18,832	18,050	95.9	1,269	7.0	NA	NA
*E. coli metagenome*	53/0/2	5,092,009	2,084,185	40.9	260,039	12.5	240,818	92.6

*Average length of all complete genomes and assemblies from the study.

**Length of all sites that are conserved across all samples.

***Number of sites with SNPs in core genome.

^¶^Number of SNPs from coding regions.

^#^See Methods.

### High resolution *Escherichia* phylotyping using PhaME

**Figure 2:**
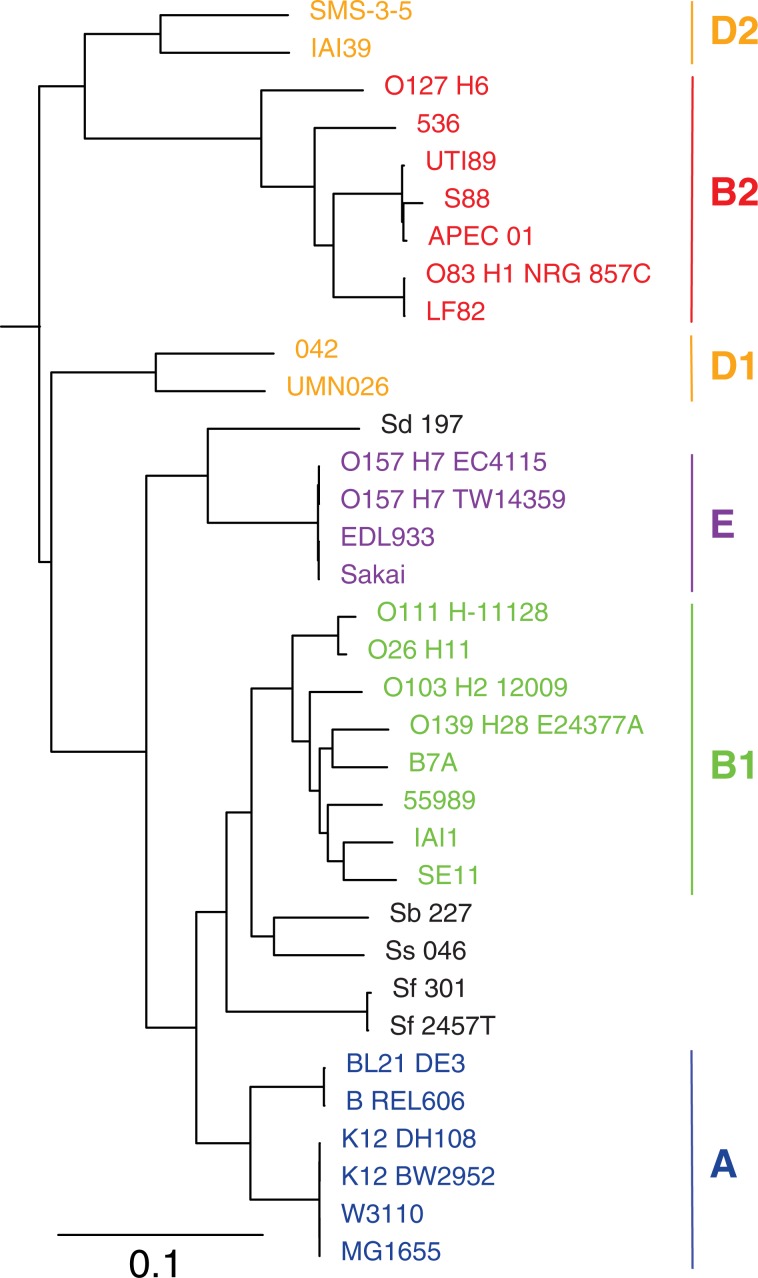
SNP based phylogeny of 35 *Escherichia* and *Shigella* genomes. All nodes have bipartition bootstrap support of 60% or greater. Clades are labeled with their corresponding *E. coli* phylogroups on the right. The tree was rooted with *E. fergusonii* ATCC 35469 as an outgroup that was removed in the figure. The scale bar indicates the number of substitutions per site.

The model bacterium *Escherichia coli* has been extensively studied, including its diversity and phylogenetic history^27^. In previous studies, phylogenetic analysis using a single gene^28^, a set of genes^27,29–31^, SNPs^32,33^, and *k*-mer profiles^34^ have consistently shown that *E. coli* strains are clustered into phylogenetic groups (A, B1, B2, D1, D2, and E) and different ‘species’ of *Shigella* also form distinct groups within the *E. coli* lineage and are not a separate genus^35^. To test whether PhaME can recapitulate the established *E. coli* tree topology, we first analyzed 35 complete genomes of *E. coli*, *Shigella*, using *E. fergusonii* as an outgroup (Table S1). PhaME detected 266,969 SNPs within the conserved core genome which consists of 2,159,296 aligned nucleotides (Table 1**)**. Similar to previously published phylogenies^27,31^, the maximum likelihood phylogeny constructed from these core SNPs grouped all *E. coli* and *Shigella* strains into their expected phylotypes (Fig. 2).

**Figure 3:**
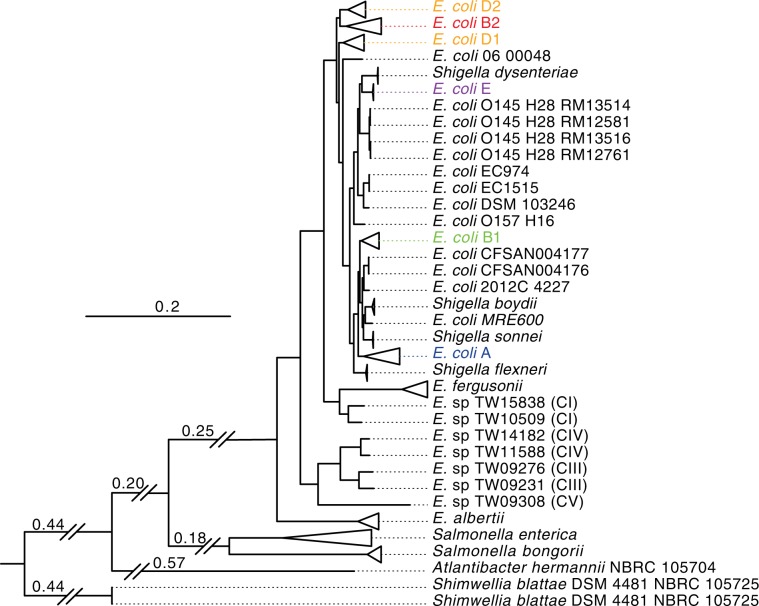
Inter-genus phylogeny using 676 *Escherichia, Shigella, Salmonella, Shimwellia, and Atlantibacter* datasets. Branches containing genomes from clades representing *E. coli* phylotypes and species with multiple strains are collapsed and labeled on the right with their corresponding phylotypes or species name. Genomes that did not form clades with any phylotypes are labeled with their full name. Genomes of cryptic *Escherichia* clades have their groups labeled in parenthesis from CI-CV. Two forward slashes in branches represents branches that were trimmed and the corresponding numbers represent the actual branch lengths. The tree was rooted with outgroup *Shimwellia* spp. The scale bar indicates the number of substitutions per site. A detailed tree that displays the names of all genomes and support values is shown in Fig. S1.

To further test PhaME’s ability to successfully group the *E. coli* phylotypes when incorporating a larger number of genomes as well as representatives of related genera, we expanded our dataset to 676 genomes. We included genomes of *Salmonella*, the incorrectly named *E. blattae* (now reclassified as *Shimwellia blattae*^36^) and *E. hermannii* (now reclassified as *Atlantibacter*^37^), several ‘cryptic clades’ of *Escherichia* that have shown inconsistent phylogenetic placement in past studies^38,39^, and additional *Escherichia* and *Shigella* datasets (Table S2). Due to the significant increase in number and diversity of genomes in this expanded dataset, PhaME detected a much smaller conserved core genome of 134,062 positions, with 40,675 SNPs (Table 1). The resulting phylogeny showed genomes from *E. coli* phylotypes and *Shigella* accurately grouped into their respective clades (Fig. 3); *Salmonella* spp. *S. bongori* and *S. enterica* were clearly distinguished and were an outgroup to all *Escherichia* (Fig. S1). This tree also resolved contested evolutionary relationships among the environmental cryptic *Escherichia* lineages. For example, consistent with the 2009 MLST study, but in contrast with the 2011 single copy core gene study, the *E. albertii* lineage diverged before *E. fergusonii*^38,39^ and *E. fergusonii* grouped with cryptic clade CI and not as an outgroup to all four cryptic clades (Fig. 3). In addition, the tree also supports reclassification and renaming of *E. blattae* to *Shimwellia blattae*^36^ and *E. hermanii* to *Atlantibacter hermanii*^37^ as these genomes clearly fell outside of *Escherichia* and *Salmonella*. In a separate naming issue, *E. fergusonii* FDAARGOS 170 (GCA_001471755.1) was placed within an *E. coli* clade. Since the construction of this tree, in its most recent assembly version in NCBI (GCA_001471755.2; May 1, 2018), it has now been reclassified as *E. coli*. PhaME was therefore able to recapitulate the established phylogeny of these related organisms, including distinguishing among *E. coli* phylotypes using hundreds of genomes from multiple genera while maintaining the internal *Escherichia coli/Shigella* topology (Fig. 3). Additionally, PhaME provides supporting evidence for reclassification of organisms that have only recently been renamed, and has helped resolve the evolutionary history among the cryptic clades of *Escherichia*.

At a granular level, we observed several additional cases of phylogenetic placement of genomes that were not in agreement with their designated species name. Four genomes annotated as *E. coli* are found with *Shigella*, namely *E. coli* MRE600^40^, *E. coli* 2012C 4227^41^, *E. coli* CFSAN004176^42,43^, and *E. coli* CFSAN004177^42^. Among these, *E. coli* MRE600 was previously shown to reside in a clade with *S. flexneri*, using a phylogeny inferred from seven housekeeping genes^40^. Our analysis instead places MRE600 as an outgroup of the *S. boydii* clade, based on core SNPs that are spread across 157 genes (Fig. S1). The other three outliers have been previously described as closely related to one another, and are known to express Shiga toxin^44^, which is consistent with the PhaME placement of this clade as an outgroup to *S. sonnei*, *S. boydii* and MRE600. Likewise, *Shigella* sp. PAMC 28760 was placed within phylotype A of *E. coli*, warranting a review of its name/description. With the rapid increase in available *E. coli* and related genomes and a shifting view of their phylogeny, we find that classic nomenclature with named phylotypes may be insufficient to categorize all new or future strains (Fig. 3). For example, four strains of *E. coli* O145 H28 form a sister clade to phylotype E and *S. dysenteriae* and do not group with any previously named phylotypes, consistent with prior observations^45^.

With the above examples, we have shown that PhaME is able to reconstruct known phylogenetic relationships using genome-wide scans for polymorphisms. The use of core genome SNPs allows for highly detailed trees capable or resolving strain to strain relationships. We have also illustrated how PhaME can help resolve long standing questions regarding species and genus-level relationships, and to better understand the granular relationship among strains, including the discovery of misnamed strains or species and potential issues with our current taxonomic nomenclature.

### *Burkholderia* phylogeny from genomes, contigs, and raw reads

**Figure 4:**
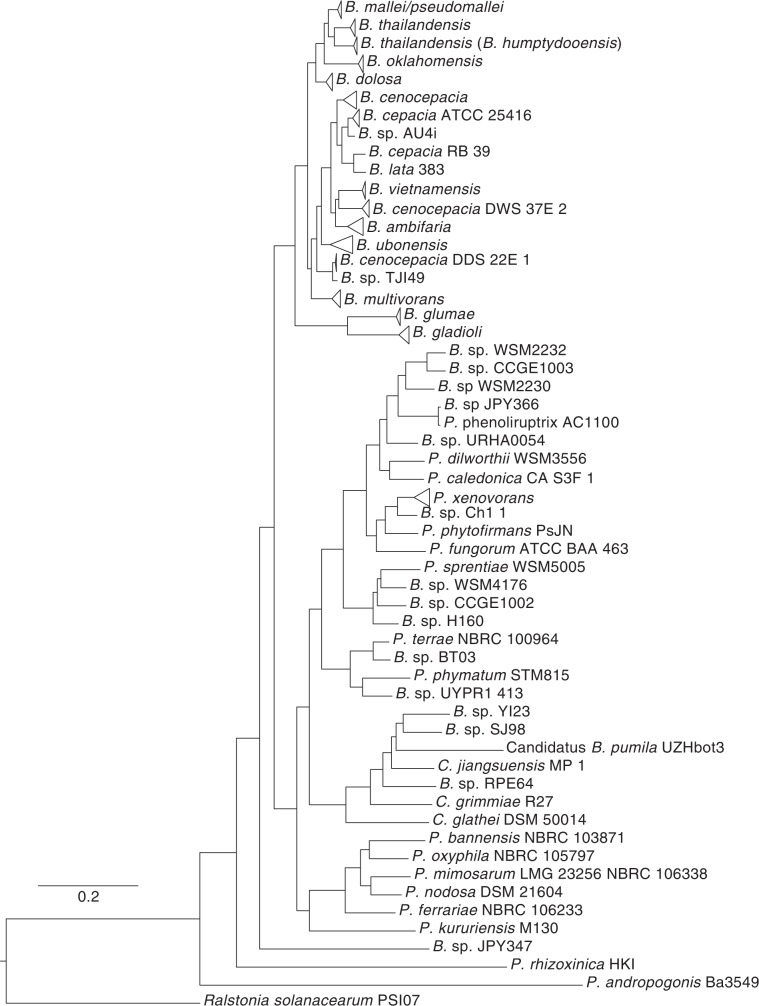
Phylogeny of *Burkholderia, Paraburkholderia, Caballeronia, and Ralstonia* using reads, contigs, and finished genomes. Maximum likelihood phylogeny from 213 samples (genomes, assemblies, and reads). Clades of the same species were collapsed and only the name of that species is shown. *Ralstonia solanacearum* PSI07 was used as an outgroup. The scale bar indicates the number of substitutions per site. A fully expanded and detailed tree can be found in Fig. S2. Detailed trees showing relationships among genomes of only the *Bc*c or within the *B. pseudomallei/mallei* group can be found in Figs. S3 and S4 respectively.

We used the large and diverse group of *Burkholderia* genomes (which have been recently divided into additional genera (*Paraburkholderia* and *Caballeronia*), to show the ability of PhaME to recreate correct phylogenies of a highly divergent set of related genomes, regardless of input data type. We used 158 complete and draft genomes and 55 raw (FASTQ) read datasets (Tables 1, S3) to infer a genus-level phylogenetic tree (Figs. 4, S2). PhaME calculated a core genome of 43,124 positions with a total of 15,180 core positions with SNPs (Table 1). The genomic plasticity of this disparate group, including genome sizes ranging from 3.6 Mbp for *P. rhizoxinica* (1 chromosome and 1 megaplasmid) (42) to 9.8 Mbp for *P. xenovorans* (2 chromosomes and a megaplasmid) (43), has contributed to the observed small core genome size. This also supports the hypothesis that *Burkholderia* are highly diverse lineage with a large ‘accessory genome’^46^ not shared among all of its members.

PhaME recapitulated all major known clades^47^ such as the *B. cepacia* complex (*Bc*c) and the *B. pseudomallei* group from the input reads, assemblies and genomes (Figs. 4, S2). While the overall topology of the tree grossly agrees with previously published phylogenies derived from concatenated housekeeping genes^47,48^, several novel observations can be made. Similar to the ribosomal protein tree^49^ but disagreeing with a 21 conserved protein tree^48^, PhaME supports the placement of the *P. kururiensis* clade as ancestral to the remaining named *Paraburkholderia* as well as the *Caballeronia* clade, bringing into question the recent renaming of *Burkholderia* into three separate genera. The PhaME tree also shows two well-supported (bootstrap value ≥60) and separate clades of *B. thailandensis* agreeing with a proposal to rename one of the clades as *B. humptydooensis* (Figs. 4, S2)^50^.

Similar to issues observed with the *Escherichia-Salmonella* phylogeny above, we also detected two *B. cenocepacia* genomes that are likely misnamed in NCBI taxonomy database (last accessed on September 26, 2019)^51^. Strain DDS 22E (GCA_000755725.1) is a close relative to the mango tree isolate *B*. TJI49^52^ in the PhaME tree, and strain DWS 37E (GCA_000764955.1) lies within the *B. ambifaria*-*B. vietnamiensis* lineage. These examples further illustrate how PhaME, using a high-resolution whole genome SNP approach, can be used to resolve disputed phylogenetic placement and nomenclature of taxonomic groups.

Since PhaME also allows the inclusion of raw read datasets into whole genome SNP phylogenies, we evaluated the accuracy of their placement compared with the assemblies and finished genomes obtained from those datasets. We found that PhaME accurately places all 55 raw FASTQ read datasets as immediate sister lineages to their respective draft assemblies or complete genomes. These results illustrate the ability of PhaME to conduct highly robust strain-level phylogenetic analysis without the need for assembly of raw sequencing data.

### Rapid reexamination of sublineages using PhaME

The *Burkholderia* genera and clades therein have been uncharacteristically difficult to discriminate using conventional polyphasic, 16 S, recA, or MLST approaches^47^. For cases like these, the ability to select a subset of genomes for analysis from within a larger phylogeny, without the need to recalculate alignments, can provide more refined insight into not only the consistency and topology within the larger tree, but can help display differences in the core genome size and the SNPs within the core. The topology of the *Bc*c subtree (Fig. S3**)** remained the same as in the larger tree with all *Burkholderia* (and renamed genera). The core genome size with only *Bc*c increased more than ten-fold to 699,313 bp and the core SNPs increased six-fold to 97,524 SNPs (Table 1). These changes did not result in topology differences but instead improved branch length resolution. Likewise, when PhaME recalculated the core genome of the highly similar *B. pseudomallei* group, the core genome and the corresponding SNPs increased by 64 and 50-fold respectively (Table 1) with no changes in the overall topology (Fig. S4). This zoomed-in phylogenetic tree also highlights the recent clonal derivation of *B. mallei* from *B. pseudomallei*, with *B. pseudomallei* 576 as the most closely related sequenced ancestor and recapitulates the paraphyletic nature of the *B. pseudomallei* strains when *B. mallei* is considered its own species^53^.These results highlight the unique functionality of PhaME to zoom into clades within a larger tree, recalculate the core genome, and rapidly generate a finer-grained phylogeny without the need to realign all the data.

### PhaME can be implemented on small eukaryotic genomes

Because PhaME can be readily applied to any taxonomic group of closely related genomes, we tested its implementation beyond bacterial lineages to larger eukaryotic genomes. Fungi are known to be a difficult group to resolve in terms of phylogenetic analysis^49^; the phylogenetic placement of fungal species displays disparities between trees based on gene sequence analyses and those based on morphological characteristics (such as modes of reproduction). This is especially true of the ‘*Saccharomyces* complex’, where the ITS regions and 26 S rDNA-based phylogenies do not show many well-supported clades^54^.

Due to the complexity and cost of assembling and finishing eukaryotic genomes, there are fewer complete genomes for many eukaryotic species. This is even the case for well-studied *Saccharomyces*, which only has 2 complete genomes. Therefore, the ability to make use of raw reads or draft assemblies/contigs can be of great value in characterizing eukaryotic genomes. We analyzed 194 *Saccharomyces* genome projects, including 7 sets of raw reads, 2 complete genomes, and 185 draft assemblies/contigs (Table S4) as input using PhaME. These datasets represent every major species from the *Saccharomyces species complex*, aside from hybrid species. PhaME calculated a core genome of 96,665 bp which consists of 24,244 SNP positions. The resulting tree topology agrees with previously published *Saccharomyces* species trees (Fig. S5)^55,56^, displaying PhaME’s ability to align and correctly recapitulate the phylogeny for small eukaryotic genomes.

A refined analysis focusing solely on the large *S. cerevisiae* clade consisting of 172 genomes increased the core genome size to 2,224,283 bp, highlighting the degree to which the core may change if a more closely related set of genomes is used, and highlights the great sequence divergence among eukaryotic species which resulted in a very small core genome size for genus-wide analysis. With such a dramatic increase in the core genome used for tree inference, one can observe much improved discrimination among strains of *S. cerevisiae*, with strong (>60) bootstrap support for most ancestral nodes (Fig. S6). This whole genome SNP analysis is a novel approach for reconstructing eukaryotic phylogenies, as the standard practice in the field is still reliant on one or several annotated genes^57,58^. PhaME can therefore provide rapid and robust discrimination among eukaryotic strains, and help better describe the relationships among closely related eukaryotic species, even when using raw read datasets.

### Using PhaME with viral samples

The *Zaire ebolavirus* outbreak that began in 2014 was rapidly characterized by large-scale sequencing and assembly of genomes from several hundred patients^59–63^ and provides a rich dataset for phylogenetic exploration. Many of the genomes and draft assemblies sequenced during the 2014/2015 *Zaire ebolavirus* outbreak, which encompassed a wide number of studies^59–63^, were recently combined into a phylogenetic study by *Dudas et al*.^64^. We used PhaME to re-analyze this dataset, and calculated 17,639 bp as the core genome size with 1,787 core SNP positions, using 1,359 *Zaire ebolavirus* genomes. The resulting PhaME tree topology is consistent with the combined maximum likelihood tree^64^, where distinct lineages are observed based largely on their geographical region of origin (Fig. S7)^59–63^.

Outbreaks such as this 2014–2015 *Zaire ebolavirus* scenario provide real-world situations where assembly of genomes is often the first step for epidemiological analysis. However, obtaining pure isolates for genome assembly is often difficult or time consuming, and assembly from metagenomic data can result in poor assembly, particularly if the target organism is not dominant or well represented in the sample. Since PhaME can accurately place raw reads in a phylogeny (as shown above for pure cultures/isolates) and because it directly aligns reads to a reference genome, it can potentially provide targeted phylogenetic analyses of an organism present within complex samples. We therefore tested PhaME’s ability to accurately place a known infectious agent within a phylogeny using reads derived directly from clinical samples.

For detailed analysis of the placement of read datasets in a phylogenetic context, we focused our analysis on viral genomes isolated from Sierra Leone. In addition to 1,031 genome assemblies, we included 93 raw read datasets that covered 99% of the *Zaire ebolavirus* genome, resulting in a 18,050 bp core genome, with 1,269 core SNP positions (Tables 1, S5). These 93 raw read datasets were quite different from one another with respect to dataset size (from 30MB to 1.2GB), average depth of *Zaire ebolavirus* genome coverage (16× to 24,204×), and percentage of *Zaire ebolavirus* reads (0.21% to 99.88%) within the sample. Regardless of these differences and the abundance of *Zaire ebolavirus* reads, the PhaME tree placed 89/93 (96%) of the raw read datasets within the same branch as the sample-matched assembled genomes (Fig. S8). Compared with their respective assemblies, the variant analysis of the four remaining datasets differed by only one or two SNPs, which resulted in their slightly different placement within the tree. The SNP differences reflect existing allelic variation within the population of viruses in the samples, which can only be captured looking at the raw sequencing data, while assemblies generally reflect the consensus sequence. PhaME provides functionality to include or exclude variants based on fold coverage and proportion of reads that support the variant. These results highlight the power of PhaME to accurately phylogenetically characterize a target organism from a wide range of clinical viral samples without the need for assembly, even when it comprises only a minute fraction of a complex sample.

### Analyzing raw metagenomic reads with PhaME

**Figure 5:**
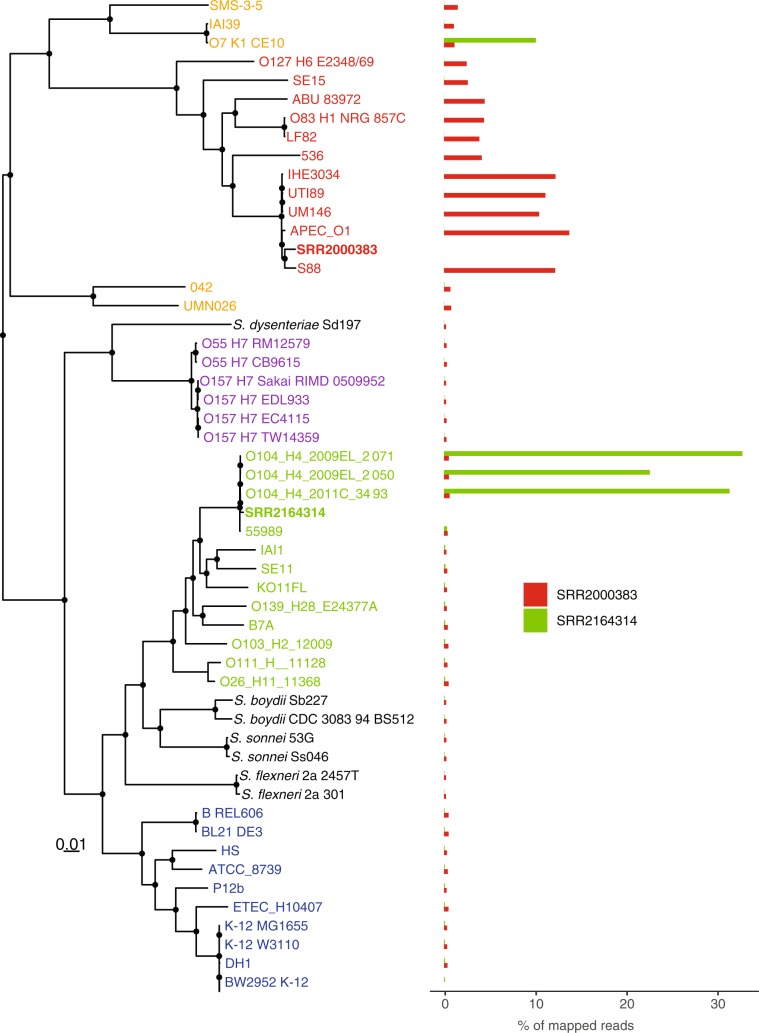
Read-based PhaME phylogenetic analysis of two human fecal metagenomics samples. Maximum likelihood tree showing 53 *E. coli* and *Shigella* genomes and the placement within the tree of the dominant *E. coli* present in the two metagenomes. The tree was rooted using outgroup *E. fergusonii* ATCC 35469. Nodes with bipartition bootstrap ≥60% are labeled with circles. The scale bar indicates the number of substitutions per site. The bar graph on the right shows the percentage of reads that mapped to each genome from the two metagenomic samples. Names of genomes are colored based on their phylotype association similar to Fig. 2.

As demonstrated with the *Zaire ebolavirus* examples, we hypothesize that a target pathogen infecting a host (assuming a mostly clonal lineage of the target organism) will be accurately placed within a phylogeny due to the read mapping and SNP calling strategy in PhaME. We further investigated fecal samples from US patients having returned from Germany during the 2011 *stx2*-positive Enteroaggregative *E. coli* (StxEAggEC) outbreak. In the context of metagenomic data, the ability to accurately phylogenetically place a target genome has two requirements: a) that a sufficient number of reads be sequenced from a target organism whose phylogeny is to be established; and b) that the target organism be a dominant clonal member of the population (including potential commensal members of that same species) in order to accurately identify SNPs belonging to the target strain. With *E. coli* as a commensal resident within the human gut, we tested the ability of PhaME to analyze fecal samples derived from two patients suspected to be infected with the 2011 StxEAggEC strain. Two fecal sample datasets (SRR2000383 and SRR2164314), each with >270 M reads, were included in a PhaME phylogeny using *Escherichia* and *Shigella* phylotype representatives **(**Table S6, Fig. 5). The target *E. coli* within the SRR2164314 fecal sample was clearly placed within the StxEAggEC phylogroup B1 outbreak strains, while the other sample was placed within a different *E. coli* clade not related to the outbreak strains (Fig. 5). These results suggest that one of the patients was indeed infected with the outbreak strain, while the other patient carried a strain from a different *E. coli* lineage.

To validate the placement of these samples within the *E. coli* phylogeny, we further characterized the metagenomes by performing taxonomy classification on the reads and also by mapping them to the human reference genome. While only the SRR2000383 sample had a strong human signal (95.73%), the majority of the bacterial hits within both samples was *E. coli*, followed by a list of other enterics common in gut microbiomes (e.g. *Eubacterium rectale*, *Enterococcus faecium*, *Lactococcus spp*., *Bacteroides spp*., etc.; Table S7). We also independently mapped the metagenome reads from both samples to their best match among all the reference genomes used in the PhaME tree, in order to evaluate the distribution of reads among the genomes. In total, 68.23% of the reads from SRR2164314 and only 0.77% of SRR2000383 mapped to the *E. coli* genomes used in the PhaME tree. While all *E. coli* genomes recruited some reads from the metagenome datasets, the dominant signal from each sample corroborated their phylogenetic placement in the PhaME generated tree (Fig. 5). This further supports the use of PhaME to establish the phylogenetic placement of target organisms, including the ability to characterize complex human fecal microbiome samples, even when in the presence of host signal, other microbial community members, and also the conflicting presence of less abundant commensal strains of the same species.

### Detecting signs of positive selections

Identifying SNPs found in coding regions enables further molecular evolutionary analyses as a post-phylogeny option that is provided in PhaME. By default, PhaME will use the HyPhy program with the Adaptive Branch-Site Random Effects Likelihood (aBSREL)^65,66^ model for detecting episodic diversifying selection on genes containing at least one SNP. Using the reference *E. coli*-*Shigella* tree (Fig. 1), we tested the application of molecular evolutionary analysis within PhaME. A total of 1387/4388 genes were found to contain at least one SNP, of which 52 genes showed statistically significant evidence of positive selection (Table S8). Among these, 37 genes showed a single lineage under positive selection, while one gene (OmpA) showed signs of positive selection in 12 lineages. OmpA is an outer member protein that is usually abundantly found on the outer surface of the cell and plays an important role in pathogenesis through its contribution to adhesion, invasion, intracellular survival, and evasion of host defenses^67^. As this protein is consistently interacting with the host, *E. coli* OmpA has been previously shown to be under strong positive selection^68^. A more detailed analyses will be required to further characterize such signals of positive selection. Among phylogenetic tools that analyze genomes, this analytical feature is unique to PhaME and allows users to explore the evolution of organisms of interest beyond simple phylogenetic trees.

### PhaME accessibility and performance

PhaME can be used on a wide variety of computing platforms from laptops with Mac OSX to Linux servers with multiple processors. Its source code is freely available in GitHub (https://github.com/LANL-Bioinformatics/PhaME) and can be installed for command line access with Bioconda (https://anaconda.org/bioconda/phame). PhaME can also be rapidly installed as a Docker container which supports use via command line, and also provides a web interface (Fig. S9) through which users can select data files, run jobs and view PhaME results (instructions at https://phame.readthedocs.io/). An example of the PhaME web interface is also hosted at https://www.edgebioinformatics.org/^69^ for use by the community.

In terms of PhaME performance, the overall computing load increases with the number and size of genomes, amount of alignments (to find the core genome), the number of SNPs, and the number of genes included in the molecular evolutionary analysis. We evaluated the wall clock time performance of PhaME to complete the full or partial analytical workflow **(**Table S9**)** using genomes of *Escherichia* and *Shigella* (Table S6) and a metagenome dataset (SRR2000383). Because of PhaME’s flexibility in terms of processing raw data or using previously aligned data, we examined the performance of various components separately. We tested the performance of generating the core genome and SNP matrix after conducting all possible pairwise alignments of 53 complete genomes. PhaME took 27.8 hours using a single processor, or 2.7 hours when increasing the number of threads and processors to 16 (see Methods for details; Table S9). Performing all pairwise comparisons is a computationally demanding task, which is why PhaME can, as an alternative, pick a single reference based on smallest average MinHash distance which approximately represents *k-mers* that are shared between two genomes^70^. The performance with the same aforementioned dataset was assessed using this MinHash-based approach for the pairwise comparison step, and using FastTree to create a phylogeny. This option reduced the runtime to 1.5 hours using a single processor and 36 minutes using 16 processors. Because PhaME also allows the addition of new datasets to be added to an existing tree (SNP matrix), we evaluated the addition of a single raw read dataset (62GB, 317 M reads) to the 53 genome SNP matrix using PhaME, which performs read mapping, variant calling, and extraction of SNPs. The process took 4 hours using a single processor. A full-fledged PhaME analysis with the 53 genomes, including MinHash-based reference selection, pairwise alignments, SNP extraction, RAxML phylogeny inference, along with molecular evolution analysis with HyPhy, took 15.16 hours to complete with 32 processors. Additional performance tests can be found in Table S9.

## Conclusions

With the rapidly growing number of available genomes and NGS read datasets, it is becoming increasingly important to have holistic yet modular analysis tools that can deal with common sequencing outputs, such as complete genomes, assembled contigs, and raw sequencing data in a standardized fashion. It is also pertinent that tools are capable of accommodating a wide variety of research goals and applications, while catering to the needs of biologists without substantial bioinformatics background or training. Here, we described a new Phylogenetic and Molecular Evolutionary analysis package, PhaME, that can rapidly process hundreds of genomes and/or raw reads from organisms across the tree of life, that produces highly robust whole genome SNP phylogenetic trees, and that can additionally estimate selective pressure in core genes along lineages of the tree. PhaME is a unique phylogenetic tool that can correctly and quickly place raw sequencing data into phylogenetic context without the need for assembly, can zoom into select lineages for rapid reanalysis of a subset of genomes, and can incrementally add samples to previously analyzed datasets. While the full functionality of PhaME can be accessed through the command line, we have implemented an easy-to-use web-based interface that can accommodate biologists with a range of bioinformatics expertise. While phylogenetic analysis has traditionally required annotated genes, PhaME represents an automated workflow for today’s genomics era that enables computing the core whole genome alignment, phylogenetic trees, and molecular evolutionary analyses within a single tool.

## Materials and Methods

### PhaME overview

We present a tool for Phylogenetic and Molecular Evolution Analyses (PhaME) that can take raw NGS reads or assembled contig(s) that represent draft or complete genomes, will align the sequences to find conserved ‘core’ sections among the input genomes, identify all SNPs (in coding and non-coding regions of the genome), infer a phylogeny, and perform evolutionary analyses to identify signals of selective pressure in genes with SNPs. PhaME is primarily written in Perl incorporating several open source software packages including the BBMap v37.66^71^ for MinHash distance calculations, MUMmer package with nucmer v3.1^72^ for genome alignment, Bowtie 2 v2.1.0^73^ or BWA v0.7.17 for read mapping, SAMtools v1.6^74^ and BCFtools v1.6 for parsing mapped reads and calling SNPs, RAxML v8.2.10^75^, FastTree v2.1.10^76^, or IQ-TREE v1.5.5^77^ for reconstruction of phylogenetic trees, and HyPhy v2.3.11^65^ or PAML^78^ for molecular evolution analyses. The overarching architecture of the PhaME analysis workflow is outlined in Fig. 1 and all steps are explained in detail in both the Supplementary Methods and online documentation at https://phame.readthedocs.io. All of the analyses were performed using PhaME v1.0.4 (DOI: 10.5281/zenodo.3458556).

PhaME can be used both via a command line interface and a web-based interface (Fig. S9). For command line use, PhaME can be installed using the source code from GitHub, or as a Bioconda package^79^. Detailed instructions on installation and for the GUI can be found on the GitHub page as well as in the online documentation at http://phame.readthedocs.io. Alternatively, we provide Docker containers that allow both command line use as well as an interactive web-interface that provides the ability to both submit jobs and view results. The PhaME web interface is deployed using a microservices framework in Docker containers that combines Flask (a python framework for user interfaces; http://flask.pocoo.org/), PostGREs (for user account database handling), Celery (for maintaining and executing PhaME; http://www.celeryproject.org/), and Redis (to keep track of task status; https://redis-py.readthedocs.io). After logging in, users are prompted to upload and select their input data through a web interface, select parameters using drop-down menus, and submit their jobs. Upon completion of a run, the users are emailed a link to a results page that contains an interactive tree viewer (https://github.com/cmzmasek/archaeopteryx-js) and pre-formatted tables. We have integrated PhaME as part of the EDGE bioinformatics platform^69^ and have made available a PhaME webserver at https://edgebioinformatics.org/. This online web service requires registration via an email which will enable running the PhaME workflow and keep track of projects.

After installation, PhaME requires a “control file” that provides parameter information and the location of input and output folders. An example control file is shown in Fig. S10. PhaME requires at least one reference genome, preferably a complete genome in FASTA format, consisting of one or more sequences that can be chromosomes, other replicons, contigs, etc. If molecular evolutionary analysis is desired, or if the user wishes to explore coding vs noncoding or synonymous vs nonsynonymous differences, the reference genome must have an associated annotation file (GFF or GFF3 file). Additional genomes in the form of raw next generation sequencing reads in FASTQ format (single or paired ends), or assembled contigs in FASTA format can also be included.

PhaME produces a number of output result files. The main outputs include pairwise alignment files, the final multiple sequence alignments of all positions with one or more SNPs, core genome alignment, maximum likelihood tree(s), text files summarizing the number of SNPs in pairwise comparisons between all aligned genomes, the position of SNPs in all input genomes, and information on whether these SNPs alter a codon and its associated amino acid. The molecular evolutionary analysis, when selected, are performed on each gene that contains a SNP and are presented in a series of files per gene.

### Whole genome alignment and core genome and SNP discovery from genomes, contigs, and reads

All complete genomes input into PhaME are initially subjected to self-comparisons using nucmer in order to remove duplicated regions or other highly similar ‘repetitive’ elements to avoid possible misleading alignments. The complete genomes then undergo pairwise whole genome alignment using nucmer in all combinations when the user wants to create a database for faster future analysis or wants all vs. all comparisons. Otherwise (default) only pairwise alignments against a designated reference genome is carried out. The reference genome can be specified by the user in the control file (from among the input genomes), picked randomly from the input genomes, or (default) identified using the MinHash distance calculated using BBMap v. 37.66^71^ to identify a complete genome with the shortest total distance among all input genomes. Moreover, based on the proportion of query genomes that aligned with a reference genome, users can automatically control the inclusion or exclusion of similar or divergent genomes by specifying it in “cutoff” parameter in the control file. This option also allows users to remove incomplete genomes that are not of desirable completion compared to the reference. Gap regions from the alignments (unaligned segments ≥1 nucleotide) are removed from downstream analyses. Input raw read datasets (either single or paired-end) are then aligned to the reference genome using Bowtie 2 (default parameters) or BWA MEM (default parameters). The mapping results are then parsed using SAMtools, BCFtools and Perl scripts to identify SNPs found in shared genomic locations. An orthologous SNP alignment is created for each genome, contig, and/or read set, and contains the nucleotides that are found in all genomes, and where at least one genome differs at that position. Given an annotation file in GFF or GFF3 format, the workflow can distinguish SNPs present within coding sequences (CDS) from those present in intergenic regions. The SNPs identified in the pairwise genome alignments as well as those identified using mapped reads are available as text files or vcf files (*.snps/*.vcfs). These SNP matrices allow for rapid recalculation of the core SNPs for any subset of genomes and for reconstruction of subtrees. In addition, pairwise SNP profiles for the core genome (*coreMatrix.txt) as well as for the core coding genome (*CDSMatrix.txt) and the core intergenic genome (*intergenicMatrix.txt) are also available.

### Phylogenetic reconstruction

The core genome or SNP alignment is used to construct a phylogenetic tree. If a GFF annotation file was provided, an additional tree can be generated from the subset of SNPs found only within coding sequences or only within intergenic regions. The phylogenetic trees are inferred using FastTree (default) and/or the RAxML maximum likelihood method and/or the IQ-TREE method. In the first two cases, PhaME builds the tree using General Time Reversible (GTR) model, accounting for gamma rate variation and proportion of invariable sites (-m GTRGAMMAI in RAxML). If IQ-TREE is chosen, the program picks a model that fits the data using their ModelFinder^80^. If RAxML or IQ-TREE are chosen, one can also perform a number of bootstraps (specified in the control file).

### Molecular evolutionary analyses

PhaME can automatically perform some of the basic molecular evolutionary analyses. Using the reference GFF file, all homologous genes containing SNPs are used to test for positive or purifying selection through the implementation of methods within the HyPhy (hyphy.org)^65^ or PAML^78^ packages. Both packages can test for the presence of positively selected sites and lineages by allowing the dN/dS ratio (ω) to vary among sites and lineages. The adaptive branch-site REL test for episodic diversification (aBSREL)^66^ model in the HyPhy package is used to detect instances of episodic diversifying and positive selection. If PAML is selected, the M1a-M2a and M7-M8 nested models are implemented. In the latter case, the likelihood ratio test between the null models (M1a and M8) and the alternative model (M2a and M7) at a significance cutoff of 5% provides information on how the genes are evolving. The results for each gene are then summarized in a table containing information on whether the gene is evolving under positive, neutral, or purifying selection, along with p-values. HyPhy is run with a model, which specifically looks for sign of positive selection in given sets of genes. The analysis produces a list of JSON files corresponding to each gene which can be uploaded to vision.hyphy.org/absrel for further analysis. We opted to provide PAML as an option, however we recommend using HyPhy for large projects due to its speed and concise output.

### Analysis of complete *E. coli*, *Shigella* spp. genomes

Complete genomes of *E. coli* from different phylotypes and *Shigella* spp. and *Escherichia fergusonii* were analyzed using PhaME (Table S1). Briefly, PhaME picked *E. coli* IAI1 as the reference genome based on MinHash distance and all other genomes/assemblies were aligned against the reference using nucmer. Orthologous positions were kept, the core genome was calculated, and the subset consisting of only the polymorphic sites were used to reconstruct a maximum likelihood phylogenetic tree using RAxML (GTRGAMMAI) with 100 bootstraps. *E. fergusonii* was used to root the tree.

### Analysis of *Escherichia* spp., *Shigella* spp., and *Salmonella* spp

Complete genomes of *E. coli, Salmonella*, and *Shigella* that were available during the time of analyses (assembly_summary_genbank.txt accessed June 20, 2017) including available genomes (complete or/and draft) for other species of *Escherichia* were used in the analyses. *S. enterica* CFSAN033543 was picked as the reference by PhaME based on MinHash distances and the resultant polymorphic sites were used to reconstruct a phylogenetic tree using FastTree, and was rooted with the *Salmonella* clade.

### Analysis of *Burkholderia* spp., *Paraburkholderia* spp., and *Caballeronia* spp. using PhaME

Complete, draft genomes, and raw reads of *Burkholderia* spp. including former *Burkholderia* genomes from the newly renamed genera *Paraburkholderia* and *Caballeronia* **(**Table S3**)** were analyzed using PhaME. Genomes from genera that have multiple available genomes were randomly selected to have a mixture of complete and draft genomes. *Ralstonia solanacearum* PSI07 was also included and used as an outgroup and PhaME picked *B. mallei* NCTC 10247 as the reference genome based on MinHash distances. Raw reads were first quality controlled using FaQCs v2.09^81^ and then added to PhaME analysis. Orthologous polymorphic positions were kept and used to build a maximum likelihood tree using RAxML (GTRGAMMAI) with 100 bootstrap supports.

Subsets of the genomes that belong to the *Bc*c or the *B. pseudomallei* groups were further analyzed using PhaME (Table S3). Genomes that belong to the corresponding clades were selected from the whole *Burkholderia* tree and the original alignments were used to recalculate the core genome and core SNPs, which were then used to reconstruct maximum likelihood tree using RAxML (GTRGAMMAI) with 100 bootstraps.

### Analysis of *Saccharomyces* spp

210 available complete, draft, and raw reads of *Saccharomyces* genomes were analyzed using PhaME (Table S4). Since the majority of available genomes were from *S. cerevisiae*, we randomly sub sampled those genomes so that the results were not too heavily biased with *S. cerevisiae* genomes. Although the majority of available genomes were from *S. cerevisiae*, there were genomes from most of the recognized species of *Saccharomyces*, including *S. kudriavzevii*, *S. bayanus, S. eubayanus, S. paradoxus, S. mikatae, S. pastorianus*, and *S. arboricola*. The complete genome of *S. cerevisiae* S288C was selected as a reference based on MinHash distances and all genomes were aligned to the reference using nucmer. To increase the size of the core genome while including all divergent species of *Saccharomyces*, we removed datasets that aligned to less than 15% of the reference genome. The conserved polymorphic sites were then used to reconstruct a phylogenetic tree using RAxML (GTRGAMMAI) with 100 bootstraps. Polymorphic sites were further divided into coding and non-coding regions. We also analyzed the subset of genomes that were found in the monophyletic lineage of *S. cerevisiae* using PhaME to obtain higher resolution phylogeny with the same reference that was used for *Saccharomyces* spp. analysis.

### Analysis of *Zaire ebolavirus*

1,610 *Zaire ebolavirus* genomes that were summarized in a recent overview publication was obtained from https://github.com/ebov/space-time ^64^. Only genomes that had a linear coverage of 99% or greater to the PhaME picked reference genome (Accession ID: KT725295) were kept (i.e. 18,693 bp or greater) and processed through PhaME. For the purpose of this analysis, all genomes were treated as “complete” to perform self-alignments using nucmer.

For raw reads analysis, we focused on a subset of genomes (1,031) that were isolated from Sierra Leone and added 138 randomly selected raw reads from the Sequence Read Archive (SRA) that correspond to analyzed genomes. We also included some Guinea samples (5) for rooting the tree. These raw reads and assembled genomes were analyzed together using PhaME (Table S5). Briefly, raw reads were quality controlled with FaQCs v2.09^81^ before they were mapped to reference genome (KR105277) using BWA and only samples that had a linear coverage of 99% or greater were kept to only analyze high quality genomes. To be reported as a SNP for the purpose of tree inference, the default requirement is set to 60% of the reads mapped to the SNP position must agree with the alternate allelic variant. For both analyses, the conserved orthologous positions that included monomorphic positions were then used to reconstruct a phylogenetic tree using IQ-TREE^77^.

### Analysis of metagenomes using PhaME

Two metagenomes (SRR2000383 and SRR2164314) from the 2011 German outbreak, along with a suite of *E. coli* and *Shigella* genomes (Table S6) representing all phylotypes were used as input into PhaME. Raw reads from metagenomes were first quality controlled with FaQCs v2.09^81^. A reference genome was picked based on MinHash distances, and all other genomes and the two metagenomes were aligned against it (*E. coli* str. K-12 substr. W3110). The resulting orthologous polymorphic positions were then used to reconstruct a maximum likelihood tree using RAxML with 100 bootstraps. As an orthogonal method to evaluate the placement of metagenomic data within the tree, we mapped the two metagenome datasets to all genomes used in the phylogeny. All genomes were thus concatenated into a single FASTA file, which was used to create a Bowtie 2 index and then reads from the metagenomes were mapped and the percentage of the reads (best hit) that were mapped to each genome was reported.

An additional independent analysis of the reads was undertaken to observe the broader taxonomic composition of the metagenomic samples. Briefly, the EDGE Bioinformatics platform^69^ was used to map the reads to the human reference genome to look at the contribution of host-derived data. The remaining reads were processed using GOTTCHA (version 2)^82^ to find the proportion of reads that map to taxonomically unique segments of RefSeq genomes.

### Molecular evolution analysis of *E. coli* genomes

53 genomes (Table S6) consisting of *E. coli, E. fergusonii*, and *Shigella* spp. were processed using PhaME to detect the list of genes that are evolving under positive selection, using HyPhy. Genes with at least one SNP and 0 gapped regions within them were identified, converted to amino acid sequences, aligned, and then checked for positive selection using aBSREL^66^ model of HyPhy. Because the size of the core genome decreases with the inclusion of additional genomes, the core genome becomes increasingly enriched in highly conserved genes and depleted in accessory genes making the choice of genomes to be included in PhaME analysis, a critical step for molecular evolutionary studies.

### Performance analysis of PhaME

We tested the performance of PhaME using a set of *E. coli*, *Shigella*, and *E. fergusonii* genomes (Table S6) on a dedicated server of a Dell PowerEdge R815 model with 512GB of RAM and a quad-processor AMD Opteron(tm) Processor 6376 @ 2.3 GHz with Bright Computing’s version of CentOS 7 of kernel version 3.10.0–229.el7.x86_64. Since PhaME is highly customizable and can process a wide range of genomic file types, we tested the performance of PhaME under different scenarios (Table S8**)** and reported some of the performance values using total wall clock time.

## Supplementary information

Supplementary Information .

Supplementary Tables S1-S9, .

## Data Availability

Genomes, complete and incomplete, were downloaded based on ftp addresses from the *assembly_summary_genbank.txt* file downloaded from NCBI (ftp://ftp.ncbi.nlm.nih.gov/genomes/genbank/assembly_summary_genbank.txt) (accessed June 20, 2017). Reads were downloaded from SRA database (https://www.ncbi.nlm.nih.gov/sra). GenBank accession numbers for the sequencing data and genomes used in this study can be found in Tables S1–S9. The PhaME workflow together with documentation can be found at https://github.com/LANL-Bioinformatics/PhaME. PhaME Control files that were used for the analyses can be found at https://github.com/mshakya/PhaME-manuscript-data (10.5281/zenodo.3610728).
