# Supplementary material for: Standardized phylogenetic and molecular evolutionary analysis applied to species across the microbial tree of life
Source: Sci Rep. 2020 Feb 3;10:1723. doi: 10.1038/s41598-020-58356-1 (PMC6997174; doi:10.1038/s41598-020-58356-1)
Supplement: Supplementary file 1 — Supplementary Information . [file 41598_2020_58356_MOESM1_ESM.pdf]

Standardized phylogenetic and molecular evolutionary analysis applied to species across the microbial tree of life

Migun Shakya[1*], Sanaa A. Ahmed[1], Karen W. Davenport[1], Mark C. Flynn[1], Chien-Chi Lo[1], and Patrick S. G. Chain[1*]

[1]Bioscience Division, Los Alamos National Laboratory, MS-M888, Los Alamos, NM 87545

*Corresponding authors: pchain@lanl.gov, migun@lanl.gov

Supplementary Methods

1. PhaME: Under the hood

PhaME's input consists of a set of genomes in fasta and/or fastq formats, and corresponding annotation files in gff3 format if downstream analyses will include coding regions.
A detailed step by step explanation of how PhaME analyzes genomes is provided below.

1.1 Selecting Reference genome

Since PhaME is a reference genome-based tool where all input genomes and metagenomes are aligned against a reference, the first step of PhaME's analysis is selecting a reference genome. Given a set of genomes (in a folder listed under the "refdir" parameter of the control file), the reference genome can be selected using one of three options: option 1- a random genome is selected from the provided set of genomes; option 2- a specific genome is selected from the set via input from the user; option 3- the MinHash distance is calculated between all genomes provided (complete genomes, draft genomes, and raw reads) to determine which reference genome has the shortest average distance to all of the other genomes. MinHash distances are calculated using its implementation in BBMap [1].

1.2 Self-nucmerization to remove repeats from reference genomes

The genome alignment portion of PhaME is built on the tool nucmer[2] for alignments of genomes in FASTA format. Each genome included is first aligned with itself using nucmer, called self-nucmerization, and then aligned regions called repeats are removed from the genomes for downstream analyses. The following nucmer command is used for the self-nucmerization step:

```
$nucmer --maxmatch --nosimplify --prefix=seq_seq ref_genomeA.fasta ref_genomeA.fasta
```

The option --maxmatch, which reports all matches, is used to ensure that all possible alignments are reported for maximal removal of repeats.

1.3 Genome Alignments

All genomes that are in FASTA format are aligned against the reference genome (see section 1.1) using following command:

```
$nucmer --maxmatch refgenome.fasta genome.fasta
```

All other options in nucmer alignments are kept at default. Some of the important ones are listed below:

-b|breaklen Set the distance an alignment extension will attempt to extend poor scoring regions before giving up (default 200)
-c|mincluster Sets the minimum length of a cluster of matches (default 65)
-D|diagdiff Set the maximum diagonal difference between two adjacent anchors in a cluster (default 5)
-d|diagfactor Set the maximum diagonal difference between two adjacent anchors in a cluster as a differential fraction of the gap length (default 0.12)
--[no]extend Toggle the cluster extension step (default --extend)
-g|maxgap Set the maximum gap between two adjacent matches in a cluster (default 90)
-l|minmatch Set the minimum length of a single match (default 20)

Also, any Ns in the genomes will not be included in the alignment.

Note: If an analysis requires running multiple iterations of PhaME on a same set of data or a subset of data, one does not need to perform the alignment over and over again. PhaME provides an option where it can keep all possible pairwise alignment of genomes from “refdir” for future analyses. All the steps mentioned in this section are the same, except that all vs. all alignment is performed compared to just one reference.

1.4 Mapping of raw reads to the reference genome

Currently, PhaME only processes short, raw reads from Illumina. If raw reads, single or paired end, are included in the analyses, they are mapped to the reference genome using either bowtie2 or BWA based on users’ input. For reads mapping to the reference genome, the following commands are used:

First, it indexes the reference genome. Depending on the mapping tool selected, one of the following commands are executed:

```
$bowtie2-build refgenome refgenome
```

or

```
$bwa index refgenome
```

The raw reads are then mapped to the reference genome using one of the following commands, depending on the mapping tool selected and whether reads are single or paired.

For bowtie2 and paired reads:

```
$bowtie2 -a -x $refgenome -1 read1 -2 read2 -S paired.sam
```

The option -a reports all possible alignments.

For bowtie2 and single end reads:

```
$bowtie2 -a -x $refgenome -U read -S single.sam
```

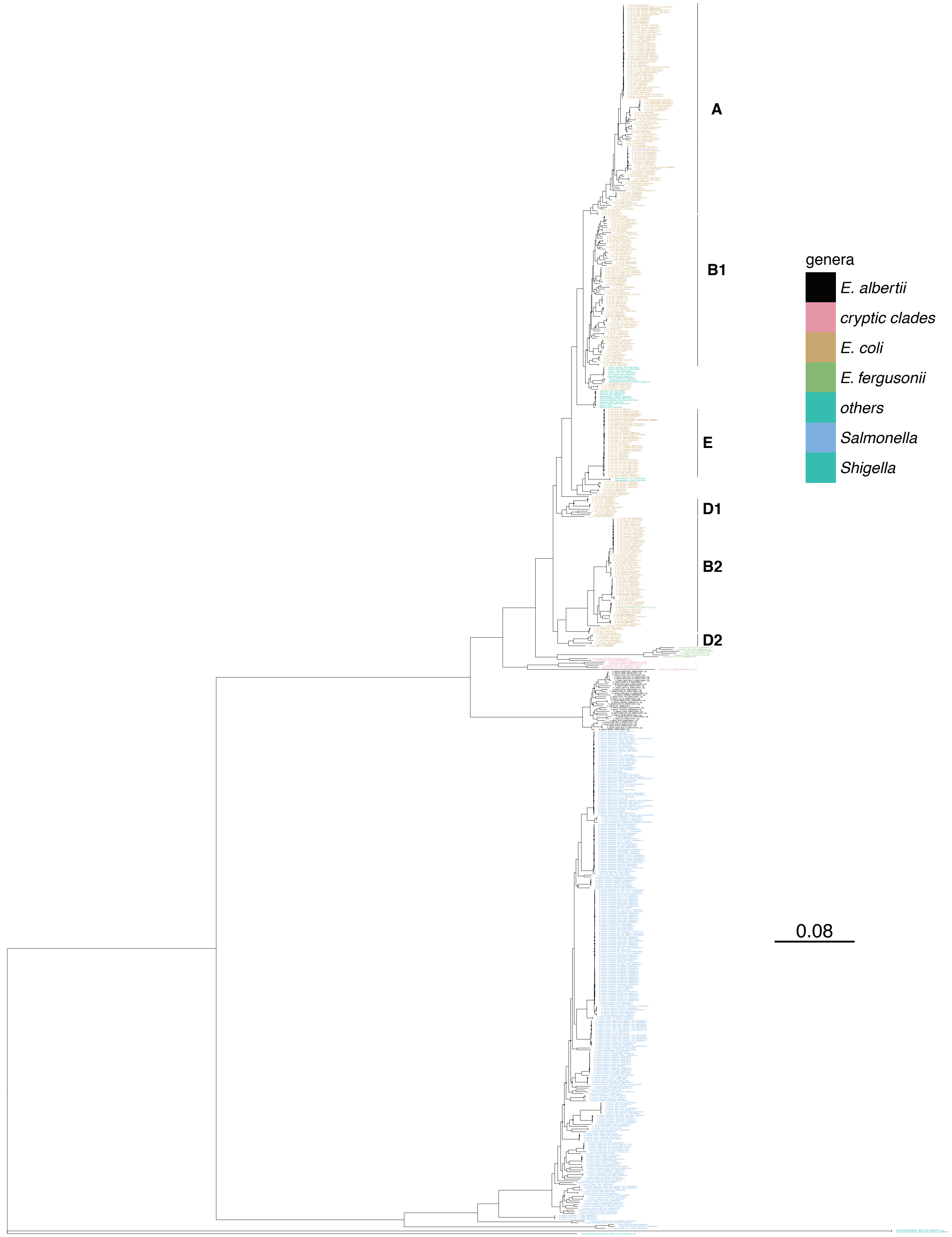

Fig. S1: **Full *Escherichia, Shigella, Salmonella, Atlantibacter*, and *Shimwellia* phylogeny.** PhaME was used to identify SNPs and reconstruct a phylogeny using FastTree for 676 genomes. Nodes with support values as calculated by FastTree to be less than 0.6 are indicated with filled circles. Genomes that are contigs are labeled with suffix "_ctg". Cryptic lineages of genus *Escherichia* are labeled with their group name in their suffix. *E. coli* phylotypes are labeled with lines on the right. The scale bar indicates the number of substitutions per site.

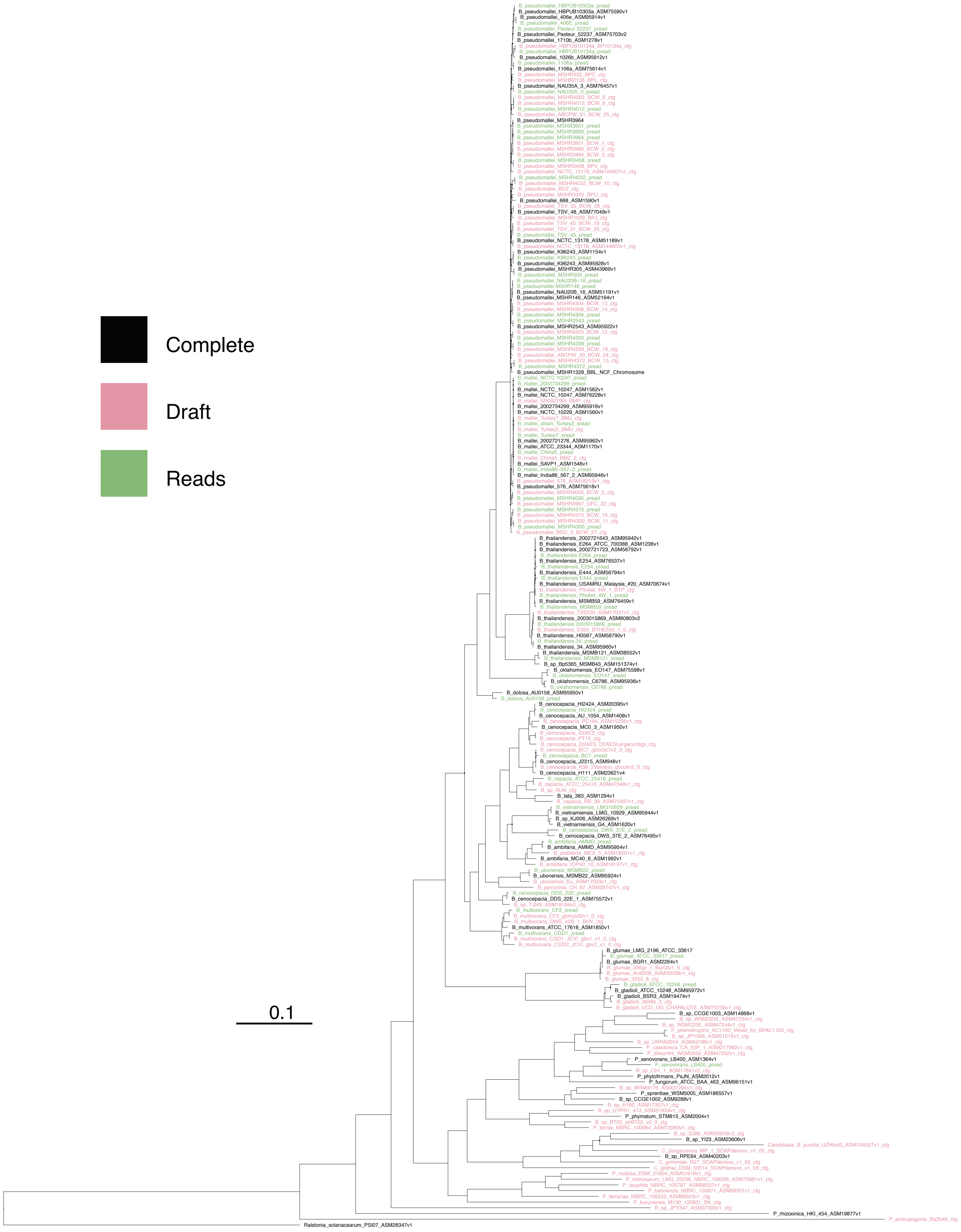

Fig. S2: **Full *Burkholderia, Paraburkholderia, Caballeronia,* and *Ralstonia* phylogeny from reads, contigs, and full genomes.** PhaME was used to identify SNPs and reconstruct phylogeny for 213 genomes using RAxML with 100 bootstraps. Nodes with bootstrap support of <60 are labeled with filled circles. The scale bar indicates the number of substitutions per site. *Ralstonia solanacearum* PSI07 was used as an outgroup.

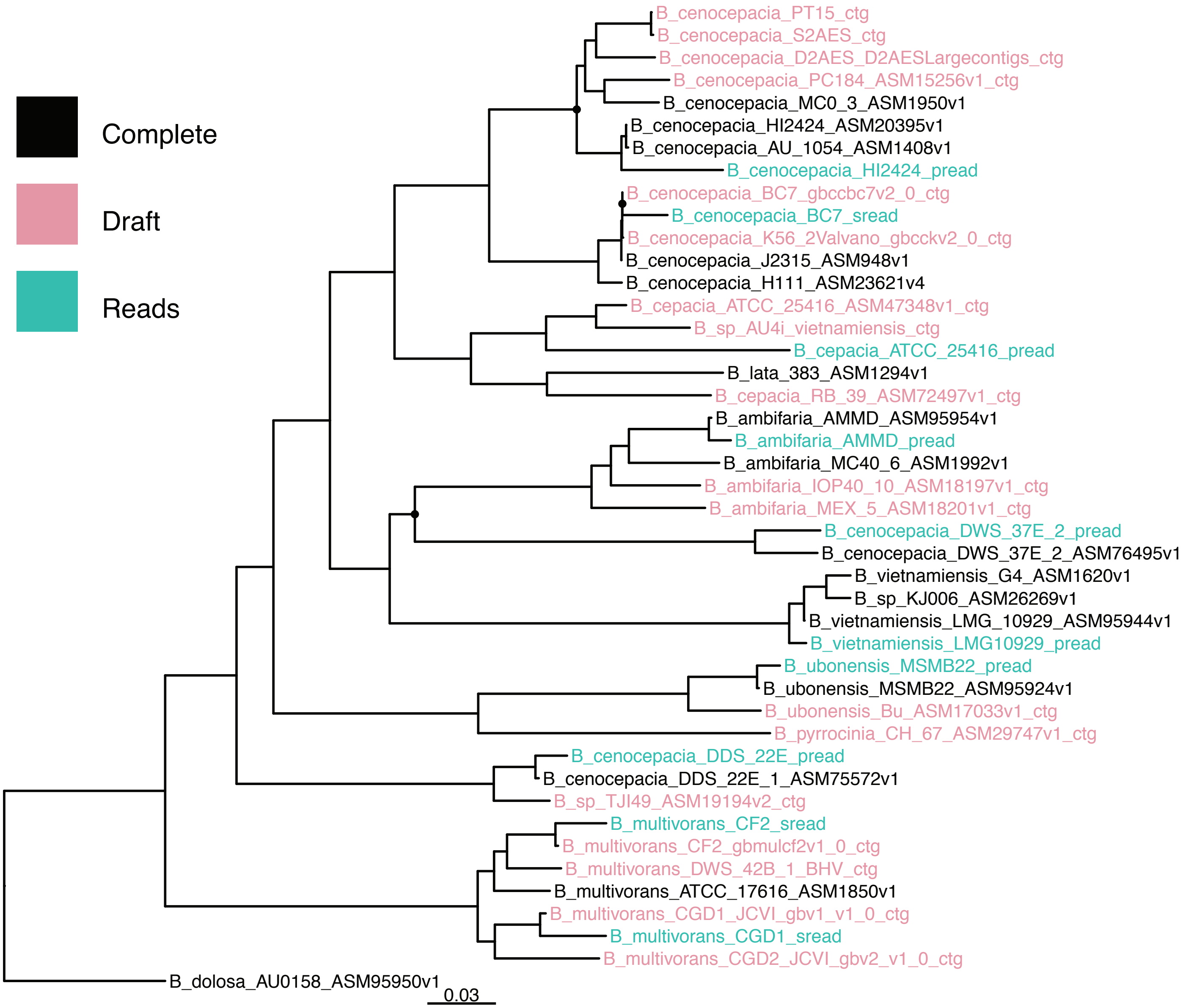

Fig. S3: **Full Phylogeny of the *Burkholderia* cepacia complex (Bcc)**. PhaME was used to reconstruct a zoomed-in subtree of 44 genomes from the larger *Burkholderia* phylogeny (**Fig. S2**) by selecting organisms in the Bcc clade. Nodes with bootstrap support of <60 are labeled with filled circles. The scale bar indicates the number of substitutions per site. *B. dolosa* AU0158 was used as an outgroup.

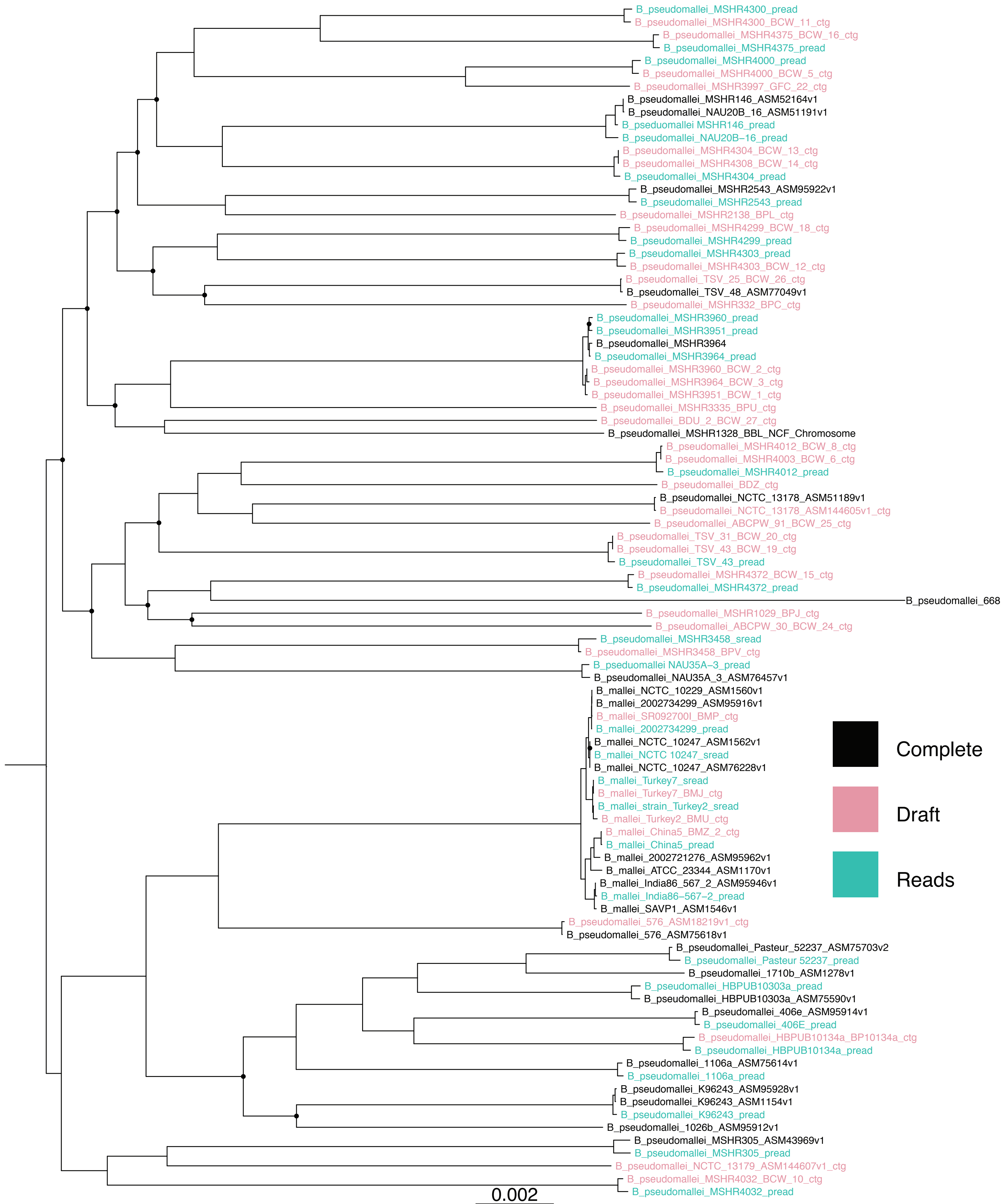

Fig. S4: **Phylogeny of *Burkholderia pseudomallei/mallei*.** A more detailed RAxML phylogeny of 94 genomes that includes *B. pseudomallei* and *B. mallei* lineages was investigated using PhaME by selecting this clade together with *B. thailandensis* E264 (as an outgroup) from the larger *Burkholderia* phylogeny (**Fig. S2**). Nodes with bootstrap support of <60 are marked with filled circles. The scale bar indicates the number of substitutions per site. The outgroup branch was removed for clarity.

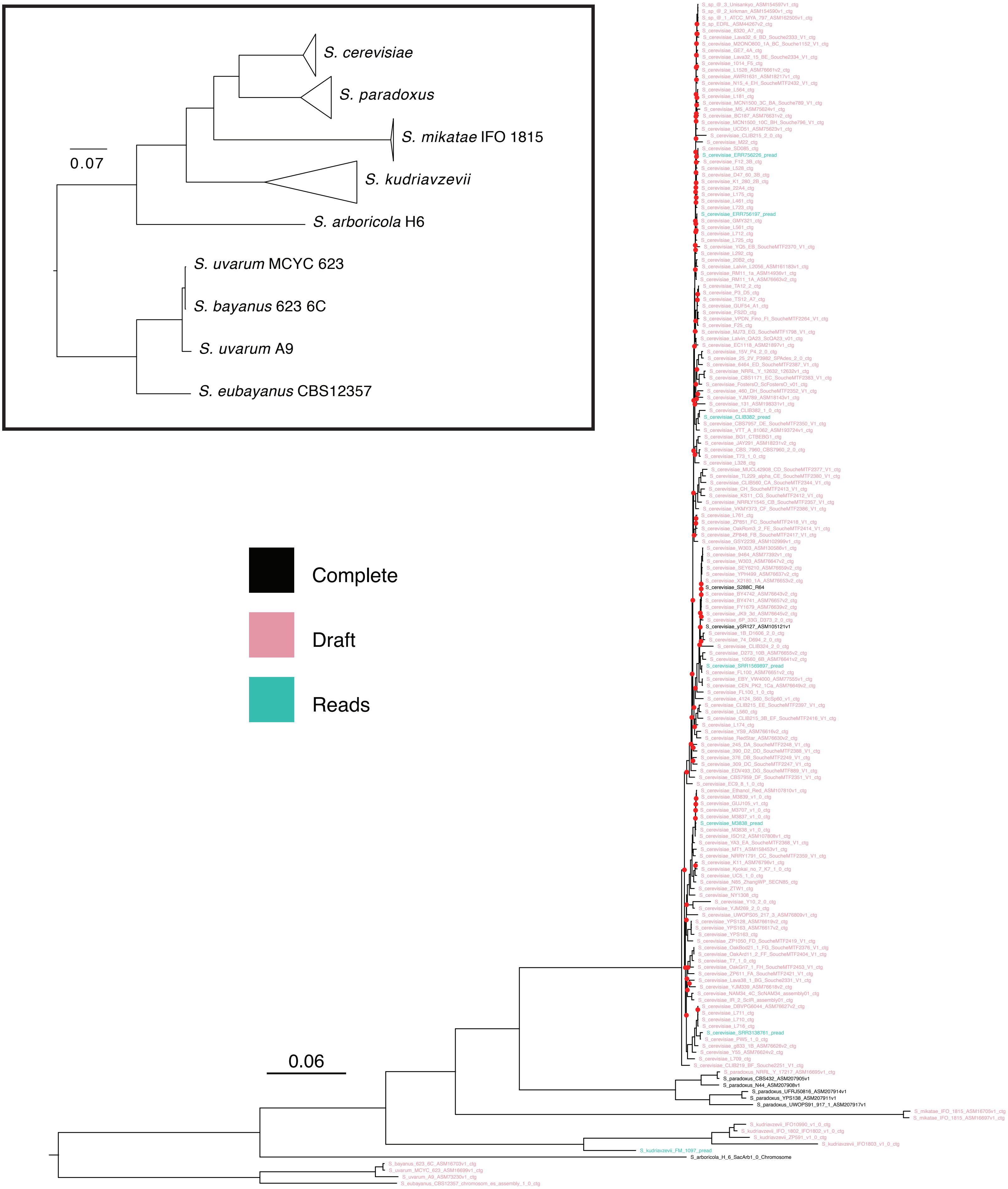

Fig. S5: **Full phylogeny of *Saccharomyces***. PhaME was used to identify SNPs and reconstruct phylogeny for 194 genomes. Nodes with bootstrap support < 60 are labeled with filled red circles. The scale bar indicates the number of substitutions per site. Inset figure shows the same

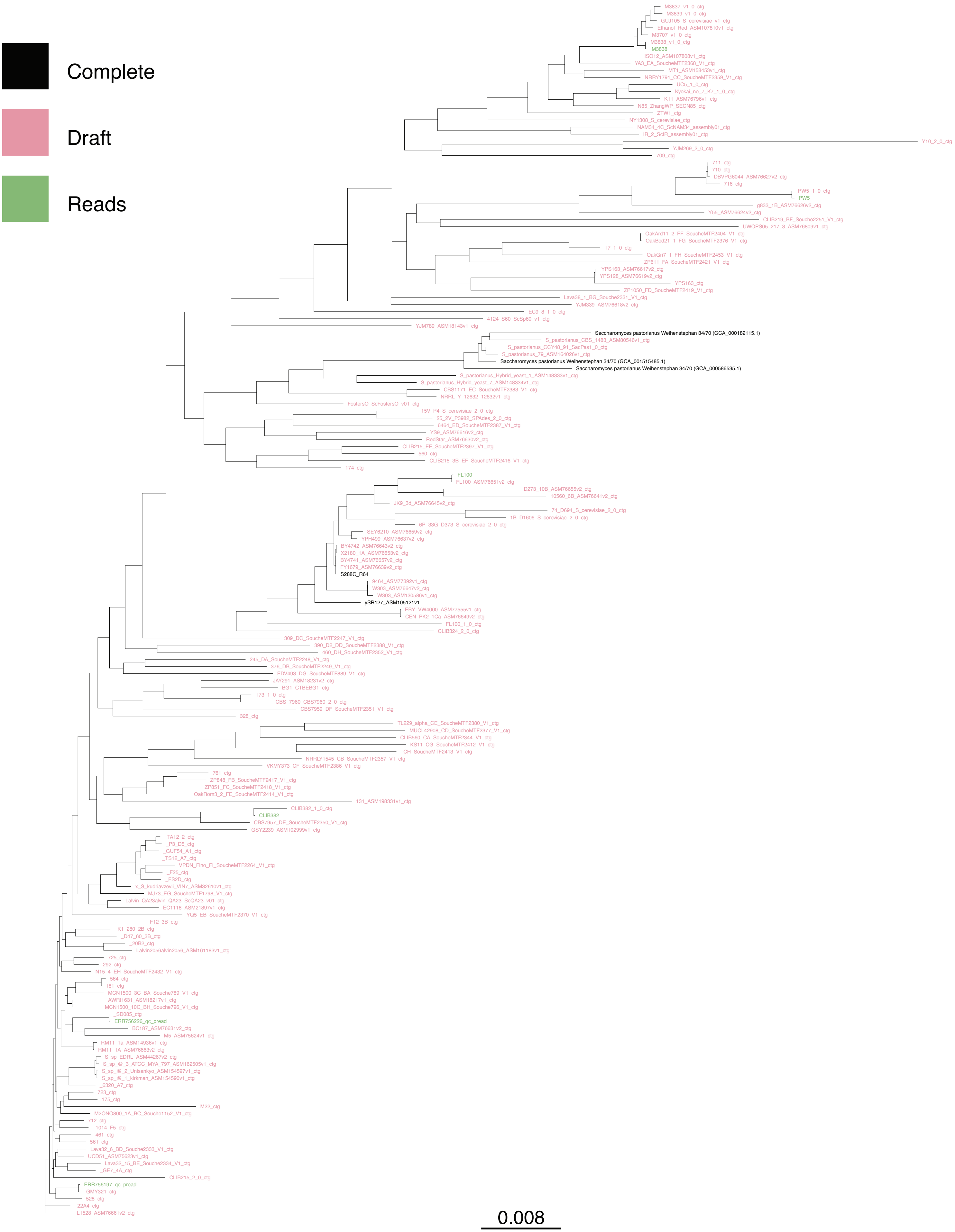

Fig. S6: **Phylogeny of *Saccharomyces cerevisiae* genomes.** PhaME was used to reconstruct *Saccharomyces cerevisiae* species phylogeny with 172 genomes by subselecting the *S. cerevisiae* species lineage from the larger genus PhaME tree (**Fig. S5**). Nodes with bootstrap support of <60 are labeled with filled circles. The scale bar indicates the number of substitutions per site.

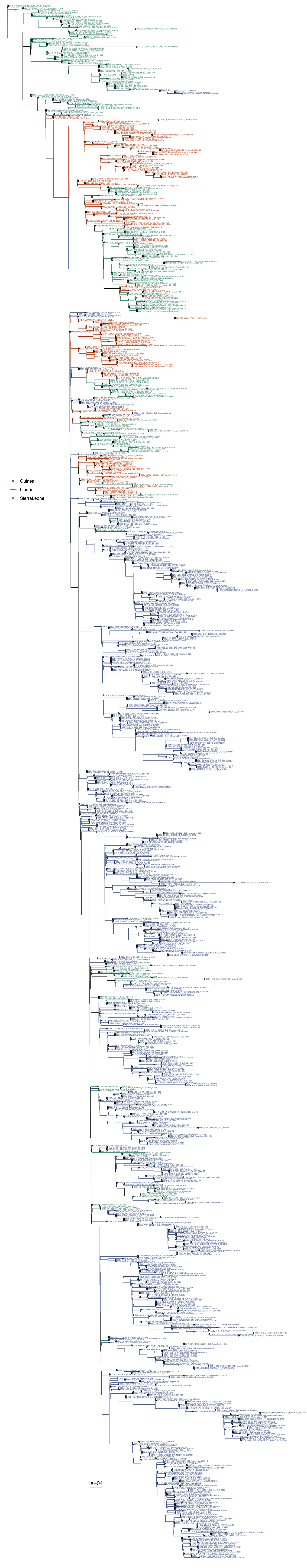

Fig. S7: **Whole genome phylogeny of *Zaire ebolavirus***. PhaME was used to reconstruct a complete phylogeny from Ebola virus genomes from 2014 outbreak in Guinea, Sierra Leone, and Liberia. Taxa are color coded based on the country from where the clinical samples were retrieved. The scale bar indicates the number of substitutions per site.

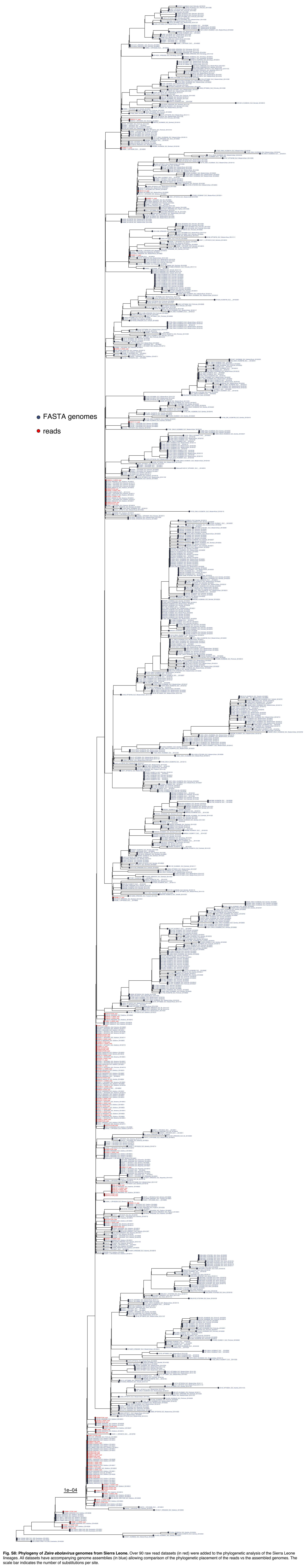

Fig. S8: Phylogeny of *Zaire ebolavirus* genomes from Sierra Leone. Over 90 raw read datasets (in red) were added to the phylogenetic analysis of the Sierra Leone lineages. All datasets have accompanying genome assemblies (in blue) allowing comparison of the phylogenetic placement of the reads vs the assembled genomes. The scale bar indicates the number of substitutions per site.

A.

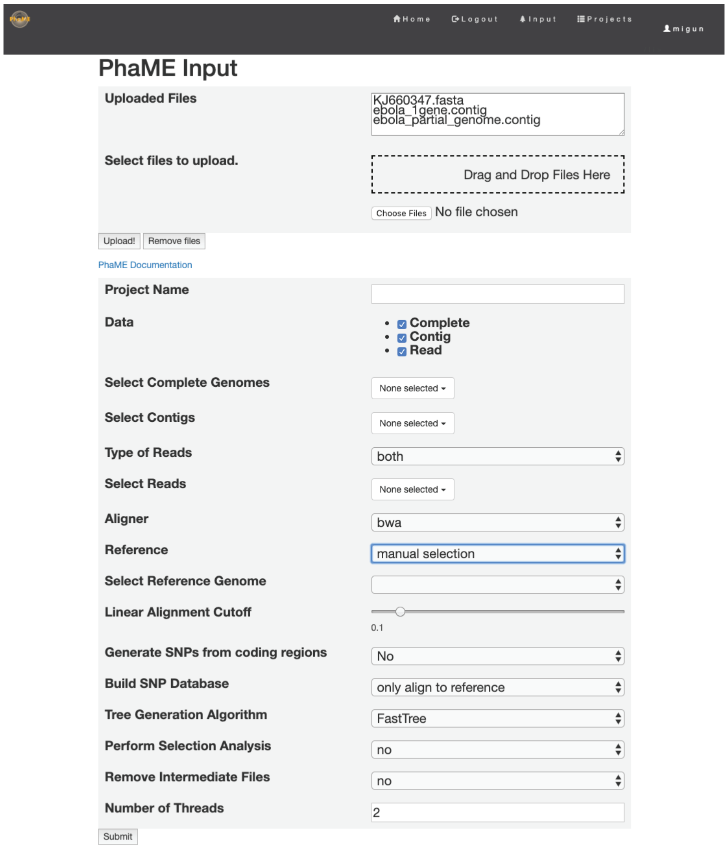

B.

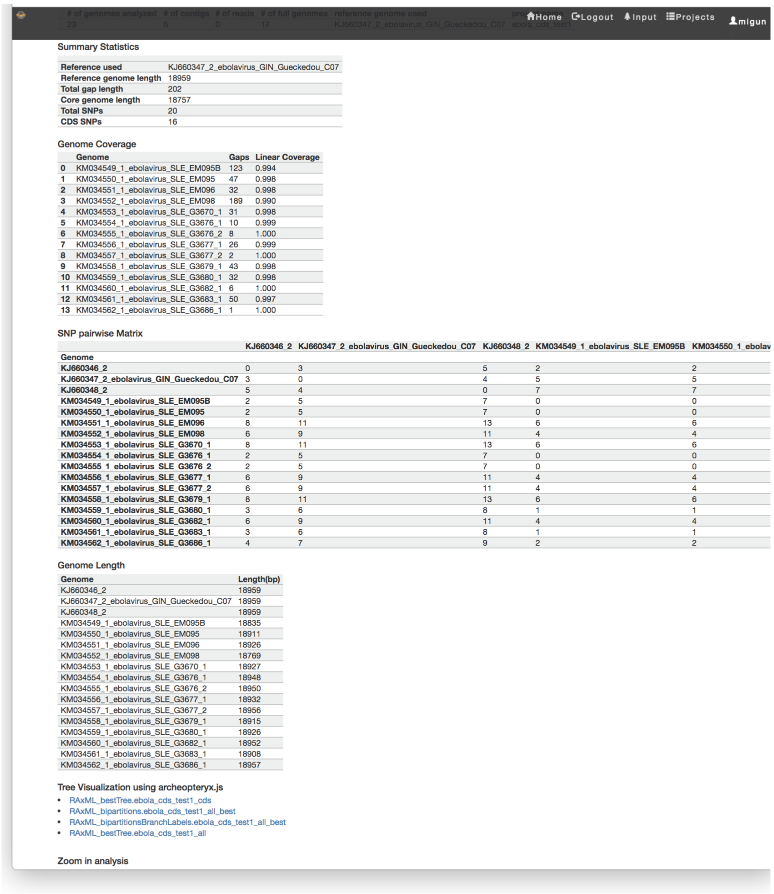

C.

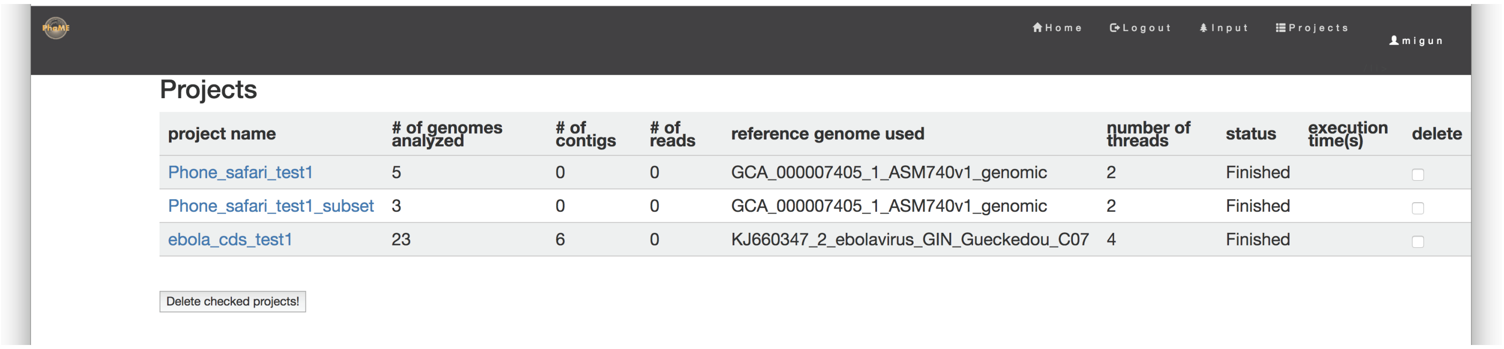

D.

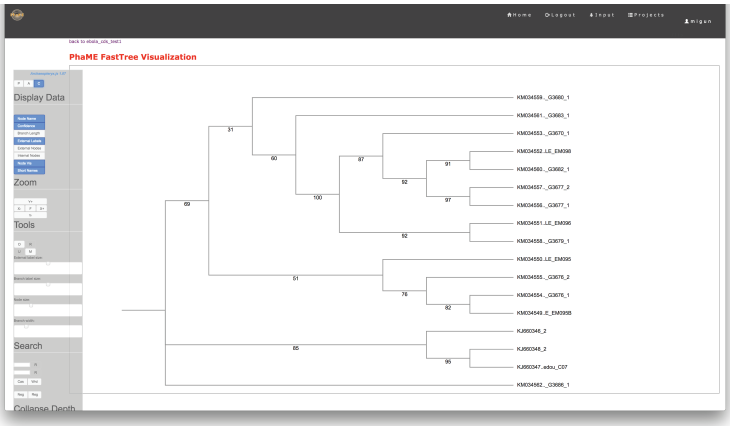

Fig. S9:Web-based user interface of PhaME. (**A**) Input page to setup PhaME run using the web-interface. (**B**) Results page showing tables with summary statistics, genome lengths, etc. (**C**) Projects page showing list of all projects that were previously run. (**D**) An interactive tree viewer (Archaeopteryx.js) implemented in PhaME.

```
refdir = refdir/ # path to directory where complete reference genomes are stored

workdir = project/ # path to directory with contigs and reads files and where output
                   # will be stored

reference = 2  # 0:pick a random reference
               # 1:use given reference
               # 2:use ANI based reference

reffile = ref.fasta  # reference filename in refdir

project = project_name  # project name, all output will have this as prefix

cdsSNPS = 0  # 0:no cds SNPs
             # 1:cds SNPs

buildSNPdb = 0 # 0: only align to reference
               # 1: build SNP database of all complete genome

SNPsfilter = 0.6 # threshold to call SNPs

FirstTime = 1  # 1:yes
               # 2:update existing SNP alignment

data = 3  # 0:only complete genomes (F); 1:only contig(C)*; 2:only reads(R)*;
          # 3:combination F+C;           4:combination F+R; 5:combination C+R*;
          # 6:combination F+C+R;         7:realignment
reads = 2 # 1:single reads
          # 2: paired reads
          # 3: both types present

tree = 0  # 0:no tree;
          # 1:use FastTree
          # 2:use RAxML
          # 3:use both
bootstrap = 0  # 0:no # 1:yes;  # Run bootstrapping
N = 100  # Number of bootstraps to run

PosSelect = 0  # 0:No; 1:use PAML; 2:use HyPhy; 3:use both

code = 1  # 0:Bacteria; 1:Virus; 2: Eukarya

clean = 0  # 0:no clean; 1:clean

threads = 2  # Number of threads to use

cutoff = 0.1  # Linear alignment (LA) coverage against reference - ignores SNPs from
organism that have lower cutoff.

* When using data option 1,2,5 need a complete reference to align/map to.
** Use data option 7 when need to extract SNPs using a sublist of already aligned
genomes.
```

Fig. S10: An example control file used for PhaME analysis.
